# Comprehensive assessment of gold nanorod-induced genotoxicity using multi-model biological systems

**DOI:** 10.1038/s41598-026-36119-8

**Published:** 2026-02-07

**Authors:** Shimaa E. Rashad, Abdelhamid A. Haggran, Ahmed Sabry S. Abdoon

**Affiliations:** 1 Microbial Genetics Department, Biotechnology Research Institute, National Research Centre, (NRC), Giza, Egypt; 2 Animal Reproduction Department, Veterinary Research Institute, National Research Centre (NRC), Giza, Egypt

**Keywords:** Salmonella typhimurium, Escherichia coli, *Saccharomyces cerevisiae* (YKO), *Liver cancer* (HepG2), Comet assay, Real-Time PCR, Bcl-2, p53, Bax genes, Biochemistry, Biological techniques, Biotechnology, Cancer, Genetics, Microbiology, Molecular biology

## Abstract

Using a variety of biological models, including human cell lines, *Salmonella typhimurium*,* Escherichia coli*, and *Saccharomyces cerevisiae* haploid knockout (YKO) strains, this study aimed to examine the genotoxic effects of gold nanorods (AuNRs). *Salmonella* and *E. coli* strains will be cultivated on LB agar plates and incubated for 16 h at 37 °C to perform bacterial tests. The cultures will be subjected to varying quantities of AuNRs after incubation. The comet test will be used to assess the degree of DNA damage in these bacterial strains. Likewise, haploid knockout strains of *S. cerevisiae* will be grown on YPD plates and incubated at 37 °C for 24 to 48 h before being exposed to different doses of AuNRs for the yeast model. Following treatment, the comet assay will also be used to evaluate DNA damage in yeast cells. The GeneMANIA platform, which offers functional association data to help the interpretation of the genetic findings, will be used to predict protein-protein interaction networks. The HepG2 liver cancer cell line’s expression levels of cancer-related genes will also be examined using real-time PCR. Particular attention was paid to the *p53*,* Bax*, and *Bcl-2* genes, which are homologous to the chosen yeast genotypes. Findings and outcomes: When compared to untreated control groups, the comet assay findings for both yeast and bacterial cells showed increased tail length, tail DNA percentage, and tail moment, indicating severe DNA damage (*P* < 0.05). According to a gene expression study, *Bcl-2* expression was significantly downregulated, whereas *p53* and *Bax* transcripts were upregulated after being exposed to AuNRs. Analysis of protein-protein interactions provided additional information about the functional arrangement of related proteins. Overall, the results indicate that gold nanorods have genotoxic qualities and lower malignant cell viability.

## Introduction

Materials engineering, environmental sciences, and medicine are just a few of the fields that have been transformed by nanotechnology. AuNRs have garnered increasing attention for biomedical applications like imaging, targeted drug delivery, photothermal therapy, and diagnostics because of their easily modifiable surface chemistry, high biocompatibility, and tunable optical properties arising from surface plasmon resonance^1,2^. Notwithstanding these benefits, there is rising apprehension about the safety of AuNRs and their possible genotoxic effects on biological systems^3,4^.

The term “genotoxicity” describes a substance’s capacity to harm genetic material, which may result in chromosomal abnormalities, mutations, or the development of cancer^5,6^. Nanoparticles’ distinct physicochemical properties such as their small size, high reactivity, and wide surface area allow them to interact in ways that are yet unclear with cellular components such proteins, membranes, and DNA^7,8^. Thus, to guarantee the safe application of AuNRs in clinical and industrial settings, thorough genotoxicity evaluations are necessary.

According to earlier research, oxidative stress and the production of reactive oxygen species (ROS), which can result in lipid peroxidation, mitochondrial damage, and DNA strand breaks, are the main mechanisms underlying the genotoxic effects of AuNRs^6–8^. These processes imply that, depending on their size, shape, and surface chemistry, AuNRs can cause both genotoxic and apoptotic reactions.

Classical microbiological systems such as *Salmonella typhimurium* and *Escherichia coli* have long been utilized in mutagenicity and genotoxicity research due to their well-characterized genetic background, simplicity, and quick growth^9,10^. Furthermore, when chosen using bioinformatics methods such as Clustal Omega based on sequence homology with human genes, YKO strains offer a eukaryotic model system for toxicological screening^11,12^. These models can be used as stand-ins for forecasting human toxicological reactions since many crucial cellular pathways are shared between humans and yeast^13,14^.

Because of their metabolic competence and importance to human health, human liver cancer HepG2 cells are commonly used in vitro model to evaluate the cytotoxicity and genotoxicity of nanoparticles^15–17^. To ascertain the apoptotic and DNA damage responses generated by nanoparticles, molecular markers like *p53*,* Bax*, and *Bcl-2* are commonly examined^18,19^. When exposed to genotoxic chemicals, changes in the expression of these genes represent cellular stress responses, such as the activation of apoptosis and the suppression of cell survival pathways^6,20^.

We used an integrated strategy in this study to assess the genotoxic effects of AuNRs in a variety of biological models, such as human HepG2 cells, yeast haploid knockout strains, and bacterial strains. Comet tests were employed to evaluate DNA damage in bacterial and yeast cells. Additionally, quantitative real-time PCR was used to assess *p53*,* Bax*, and *Bcl-2* gene expression alterations in HepG2 cells in order to clarify the molecular processes of genotoxicity. This interdisciplinary approach aids in the safe design of nanomaterials for biological applications and offers insightful information about the possible genotoxic hazards connected to AuNRs exposure.

## Materials and methods

### Synthesis of gold nanoparticles

The gold nanorods (AuNRs) used in this study were synthesized using the seed-mediated growth method described by Murphy et al. (2011)^21^. Chloroauric acid (HAuCl_4_·3 H_2_O; Sigma-Aldrich, USA) served as the gold precursor, and cetyltrimethylammonium bromide (CTAB) was used as the surfactant and stabilizing agent during both the seed and growth stages. The optical characteristics of the synthesized AuNRs were confirmed using a V-630 UV–Vis spectrophotometer (Jasco, Japan), which showed the expected strong longitudinal surface plasmon resonance peak at 808 nm and a weaker transverse peak near 530 nm. Transmission electron microscopy (TEM; JEOL JEM 2010, 200 kV) further verified the uniform rod-like morphology with an average length of 50.1 ± 8.2 nm, confirming successful synthesis and structural homogeneity^22^.

### *Salmonella typhimurium* strains and *Escherichia coli* strains are examples of bacterial strains and culture conditions

*Salmonella typhimurium* and *Escherichia coli* were the strains of bacteria employed in this investigation. At 37 °C and constant shaking (180 rpm), cultures were cultivated in Luria-Bertani (LB) broth until they reached the mid-logarithmic phase (OD_600_ = 0.5–0.6). Centrifugation at 3000 × g for 5 min at 4 °C was used to extract the bacterial cells, which were then twice washed with ice-cold phosphate-buffered saline (PBS, pH 7.4). For treatment, bacterial suspensions (1 × 10^7^ cells/mL) were exposed to AuNRs at concentrations of 0.5 mg/25 mM, 1.0 mg/25 mM, and 1.5 mg/25 mM (equivalent to 20, 40, and 60 µg/mL) for 16 h at 37 °C before proceeding to comet assay analysis. All experiments were performed in triplicate (*n* = 3).

Creating Slides, 1% normal melting point (NMP) agarose was used to pre-coat microscope slides, which were then left to air dry at room temperature. To embed cells, 100 µL of 0.7% low melting point (LMP) agarose kept at 37 °C was combined with 50 µL of the bacterial culture. The mixture was promptly applied to the slides that had already been prepared, covered with coverslips, and let to set for ten minutes at 4 °C.

After cell lysis solidified, coverslips were carefully taken off. The slides were submerged in lysis buffer (pH 10, 1% Triton X-100, 100 mM EDTA, 10 mM Tris-HCl, and 2.5 M NaCl) with 1 mg/25 mM lysozyme added. To guarantee that the bacterial cell wall was broken down by enzymes, the slides were incubated for an hour at 37 °C. To guarantee that all proteins and membrane components were removed, the slides were then placed in lysis buffer containing 0.5 mg/25 mM proteinase K and treated for the entire night at 37 °C.

In order to enable DNA unwinding and the expression of alkali-labile sites, the slides were washed with distilled water following lysis and then incubated in an alkaline unwinding buffer (300 mM NaOH, 1 mM EDTA, pH > 13) at 4 °C for 30 min in the dark. In the same buffer, electrophoresis was carried out for 30 min at 4 °C at 25 V (1 V/cm). To prevent further damage to the DNA, the electrophoresis was carried out in low light.

After electrophoresis was finished, slides were neutralized by immersing them in 0.4 M Tris-HCl buffer (pH 7.5) for two to five minutes at room temperature. SYBR Green I (1:10,000 dilution) was used to stain DNA for ten minutes in the dark. Slides were air-dried and gently washed with distilled water after staining.

Image Analysis: A fluorescence microscope with the proper filter set was used to visualize comet pictures. Utilizing computerized image analysis software at least 50 randomly chosen nucleoids per sample were examined. Standard comet metrics, such as tail length, tail DNA percentage, and tail moment, were measured in order to quantify DNA damage.

### The haploid strain of the yeast *Saccharomyces cerevisiae*

We purchased the complete set of knock-out haploid yeast strains (Mat-A) (Cat. no. 95401.H2) from the Invitrogen corporation (1800 Faraday Avenue, PO Box 6482, Carlsbad, CA, 92008, USA).

Based on the original procedure outlined by^12,23^, an in vitro Comet assay was conducted. YKO strains were treated were exposed to AuNRs concentrations of 1.0 mg/25 mM, for 24–48 h at 37 °C. Additionally, an untreated control medium devoid of chemical components was employed. One cubic centimeter of cold PBS was filled with one gram of crushed material. After five minutes of stirring, this suspension was filtered. 600 µL of low-melting agarose (0.8% in PBS) was combined with 100 µL of cell suspension.

On slides that had already been coated, 100 µL of this combination was unfolded. For fifteen minutes, the coated slides were submerged in lyses buffer (0.045 M TBE, pH 8.4) that contained two.5% SDS. The slides were put in an activity chamber with a TBE buffer that was comparable but without SDS. One hundred mA and two minutes of V/cm were the activity conditions. Ethidium bromide staining (20 µg/m1) at 4 °C. A visible radiation magnifier was used to assess the migratory patterns of polymer fragments in 100 cells for each dose level while the materials were still moist (with excitation filter 420–490 nm, issue 510 nm). To count and quantify the comet’s size, the lengths of the alien object’s tails were measured from the nucleus to the top of the tail, with a 40x increase. Gel Red-stained polymer was observed using a 40x objective on a fluorescence magnifier in order to visualize polymer damage. Comet Score software (version 2.0, TriTek Corp., USA) was used to examine comet assay photos in order to measure DNA damage metrics such as tail length, tail DNA percentage, and tail moment. Tail moment values were averaged per slide and at least fifty randomly chosen nucleoids were assessed for each sample. One-way ANOVA and Duncan’s multiple range test were used in the statistical analysis to see whether there were any significant differences between the treatment groups. Version 18.0 of SPSS software (SPSS Inc., Chicago, IL, USA) was used for all statistical analyses. The data were presented as mean ± standard error (SE), with *p* < 0.05 being considered significant.

In this study, four haploid knockout strains with entirely different genotypes were employed. Genes that matched the yeast genes used in this study were selected I (Table 1). The *Saccharomyces* Genome Database (SGD), (https://www.yeastgenome.org/).

**Table 1:** A selection of yeast strains with knockout gene.

Selected strains	Selected genes of yeast strains
YPR124W	*CTR1*
YMR192W	*GYL1*
YMR182C	*RGM1*
YMR193W	*MRPL24*

For each biological model (*Salmonella typhimurium*,* Escherichia coli*, *Saccharomyces cerevisiae* YKO strains, and HepG2 cells), this table gives a summary of the experimental setup utilized in the study, including the culture conditions, gold nanorod concentrations, exposure periods, and assay endpoints. By graphically displaying the interactions between AuNRs and each studied organism, the table aims to improve reproducibility and clarity (Table 2).

**Table 2:** An overview of the experimental setup for exposure to gold nanorods (AuNR) in all biological models.

Model organism	Culture condition	AuNR concentrations	Exposure time	Endpoint assay	Notes
*Salmonella typhimurium*	LB agar, 37 °C	0.5, 1, 1.5	2 h	Comet assay	50 nucleoids/ sample
*Escherichia coli*	LB agar, 37 °C	0.5, 1, 1.5	2 h	Comet assay	Same conditions
*S. cerevisiae* YKO strains	YPD, 30 °C	Single AuNR concentration	2 h	Comet assay	4 knockout strains tested
HepG2 cells	RPMI/DMEM, 37 °C	½ IC_50_ of G2, G3	24 h	qRT-PCR (*p53*, *Bax*,* Bcl-2*)	Gene expression

### Protein-protein interaction prediction

The communication network was used in accordance with the sequence. A versatile and easy-to-use web interface, GENEMANIA (http://www.genemania.org) is used to prioritize genes for particular studies, analyze sequence lists, and assess gene function hypotheses.

### Resources for information

Information about co-expression from the organic phenomenon Omnibus (GEO); information about physical and genetic interactions from Bio GRID; information about predicted macromolecule interactions backed by orthology from I2D; and information about pathways and molecular interactions from Pathway Commons, which combines information from Memoria, Bio GRID, and Pathway Commons.

Network of interactions between yeast proteins.

### RT-PCR quantitative analysis

The GeneJET RNA Purification Kit (Thermo Scientific, #K0731, USA) was used in accordance with the manufacturer’s instructions to extract total RNA from the human hepatocellular carcinoma cell line (HepG2), which was acquired from the American Type Culture Collection (ATCC, Manassas, VA, USA). RevertAid H Minus Reverse Transcriptase (Thermo Scientific, #EP0451, USA) was used to create complementary DNA (cDNA) from 5 µg of total RNA^24–27^. The Step One Plus Real-Time PCR System (Applied Biosystems, USA) and Maxima SYBR Green/ROX qPCR Master Mix (Thermo Scientific, #K0221, USA) were used for quantitative real-time PCR (qRT-PCR). 12.5 µL of 2× master mix, 2 µL of cDNA, 1 µL of each primer (forward and reverse), and 8.5 µL of nuclease-free water were all included in each 25 µL reaction. The initial denaturation at 95 °C for 10 min was followed by 40 cycles of 95 °C for 15 s, 60 °C for 30 s, and 72 °C for 30 s. Amplification specificity was verified by a melting curve study (63–95 °C). The 2^-ΔΔCt method was used to measure the relative expression of the *p53*,* Bax*, and *Bcl-2* genes, normalized to the GAPDH housekeeping gene. The following were the primer sequences (Table 3).

**Table 3:** Target and reference gene primer sequences are listed in.

Gene	Forward primer (5´→ 3´)	Reverse primer (3´→ 5´)
*Bax*	CCTGTGCACCAAGGTGCCGGAACT	CCACCCTGGTCTTGGATCCAGCCC
*Bcl-2*	AGGAAGTGAACATTTCGGTGAC	GCTCAGTTCCAGGACCAGGC
*p53*	TAACAGTTCCTGCATGGGCGGC	AGGACAGGCACAAACACGCACC
*β-actin*	CACCAACTGGGACGACAT	ACAGCCTGGATAGCAACG

Both the housekeeping gene and the target genes’ cycle threshold (Ct) values were noted. As explained by^6^, the 2^-ΔΔCt method was used to calculate the relative quantification of gene expression.

The ½ IC_50_ concentrations of AuNRs that were previously determined in our prior work are referred to as the G2 and G3 samples^6^. In particular, these concentrations were obtained from HepG2 cell cytotoxicity experiments, where the IC_50_ values were calculated and reported in that paper. In order to assess sublethal genotoxic and apoptotic reactions, the ½ IC_50_ dosages (G2 and G3) were chosen for gene expression analysis in the present investigation. Doxorubicin was used as a positive control to validate assay performance and ensure expected activation of apoptotic gene pathways.

The comet assay was used for evaluating genotoxic effects in bacterial, yeast, and mammalian models due to its excellent sensitivity in identifying DNA single- and double-strand breaks as well as alkali-labile sites at the single-cell level. This technique is well known for its repeatability and capacity to measure DNA damage in individual cells^4,12^. Similar to this, HepG2 cells’ expression of genes linked to apoptosis and DNA damage (*p53*,* Bax*, and *Bcl-2*) was assessed using quantitative real-time PCR (qRT-PCR)^23,24^, which provided molecular-level validation of the observed genotoxic and apoptotic responses. Every experiment was carried out in triplicate to guarantee reproducibility and statistical reliability, and all assays included untreated control groups for baseline comparison.

### Statistical analysis

Means ± standard error (SE) is used to display all data. SPSS software, version 18.0, was used to conduct statistical analyses (IBM, 2011). To identify statistically significant differences between groups, one-way analysis of variance (ANOVA) and Tukey’s post hoc test were employed, with a significance threshold of *P* < 0.05. The half-maximal inhibitory concentration (IC_50_) for cytotoxicity analysis was computed using the following formula: IC_50_ = (0.5 – b) / a, where b is the intercept and an is the slope factor, which indicates how well the biological activity was inhibited. Each reaction was carried out in triplicate for qRT-PCR (*n* = 3 independent biological replicates, each reaction technical duplicate). The 2^-ΔΔCt technique was used to determine relative gene expression levels, and the normalized fold-change data was subjected to statistical analysis.

## Results

### DNA fragmentation by comet assay

**Fig. 1:**
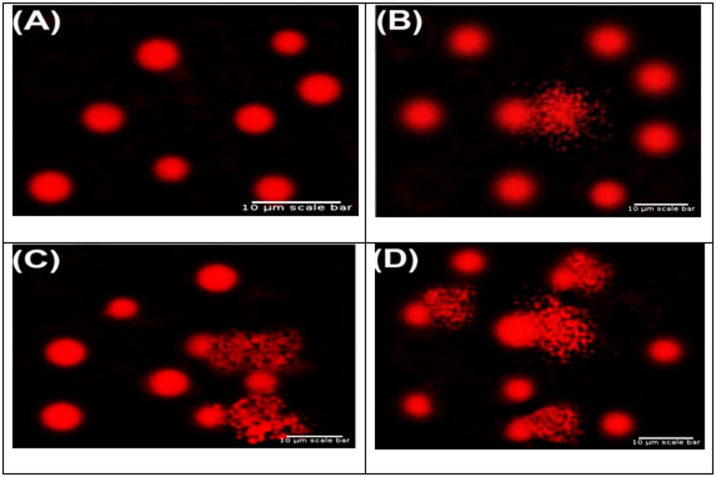
Typical comet test pictures demonstrating DNA damage in *Salmonella typhimurium* following exposure to gold nanorods at varying concentrations: (A) control, (B) Slightly damaged DNA, (C) Moderately damaged DNA, and (D) Very damaged DNA. A fluorescent microscope with a 400× magnification was used to take the pictures. 10 μm is the scale bar.

**Fig. 2:**
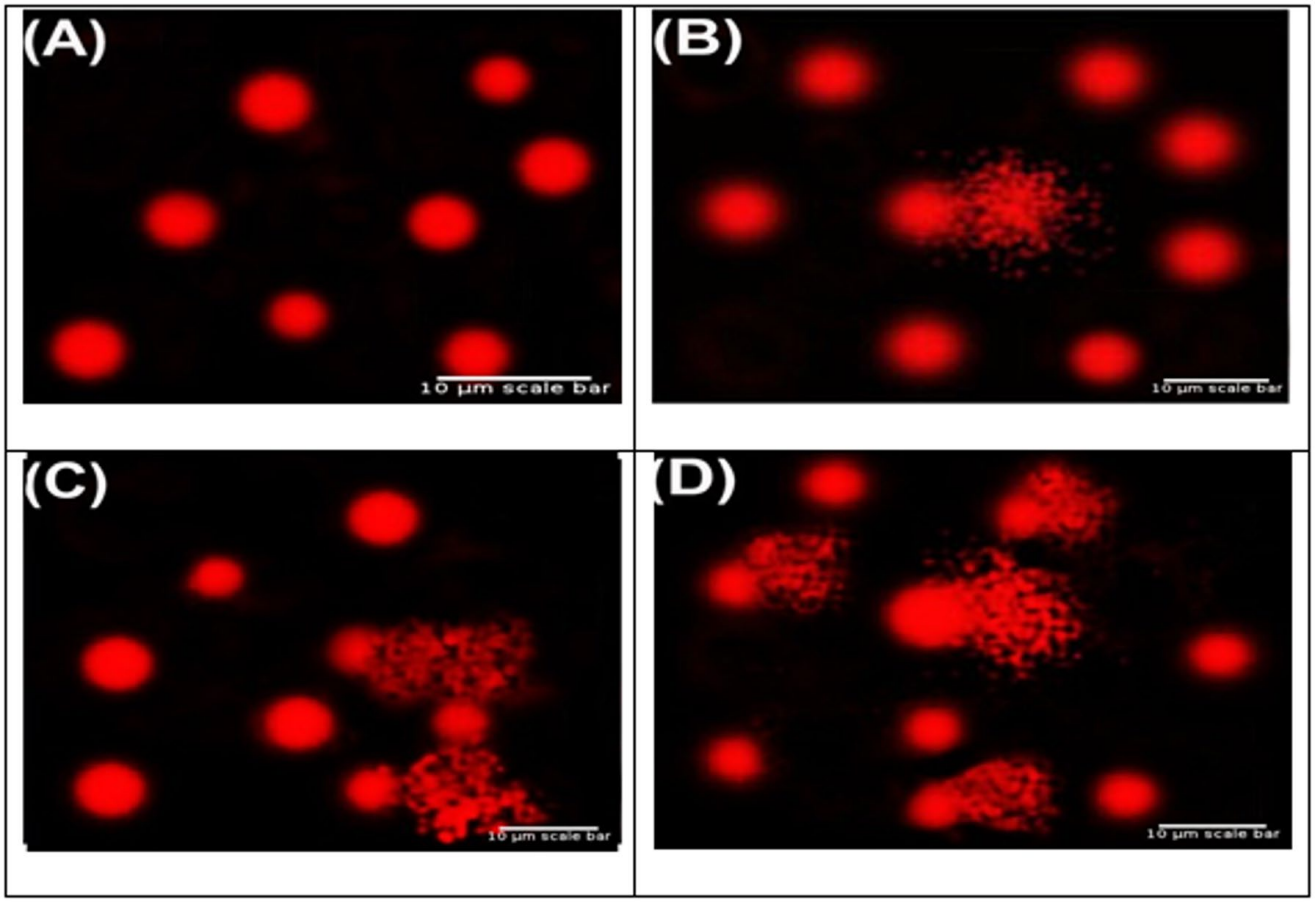
Standard comet test images showing DNA damage in *E. coli* after exposure to different concentrations of AuNRs: (A) control, (B) Slightly damaged DNA, (C) Moderately damaged DNA, and (D) Very damaged DNA. The images were captured with a 400× magnification fluorescence microscope. The scale bar is 10 μm.

**Fig. 3:**
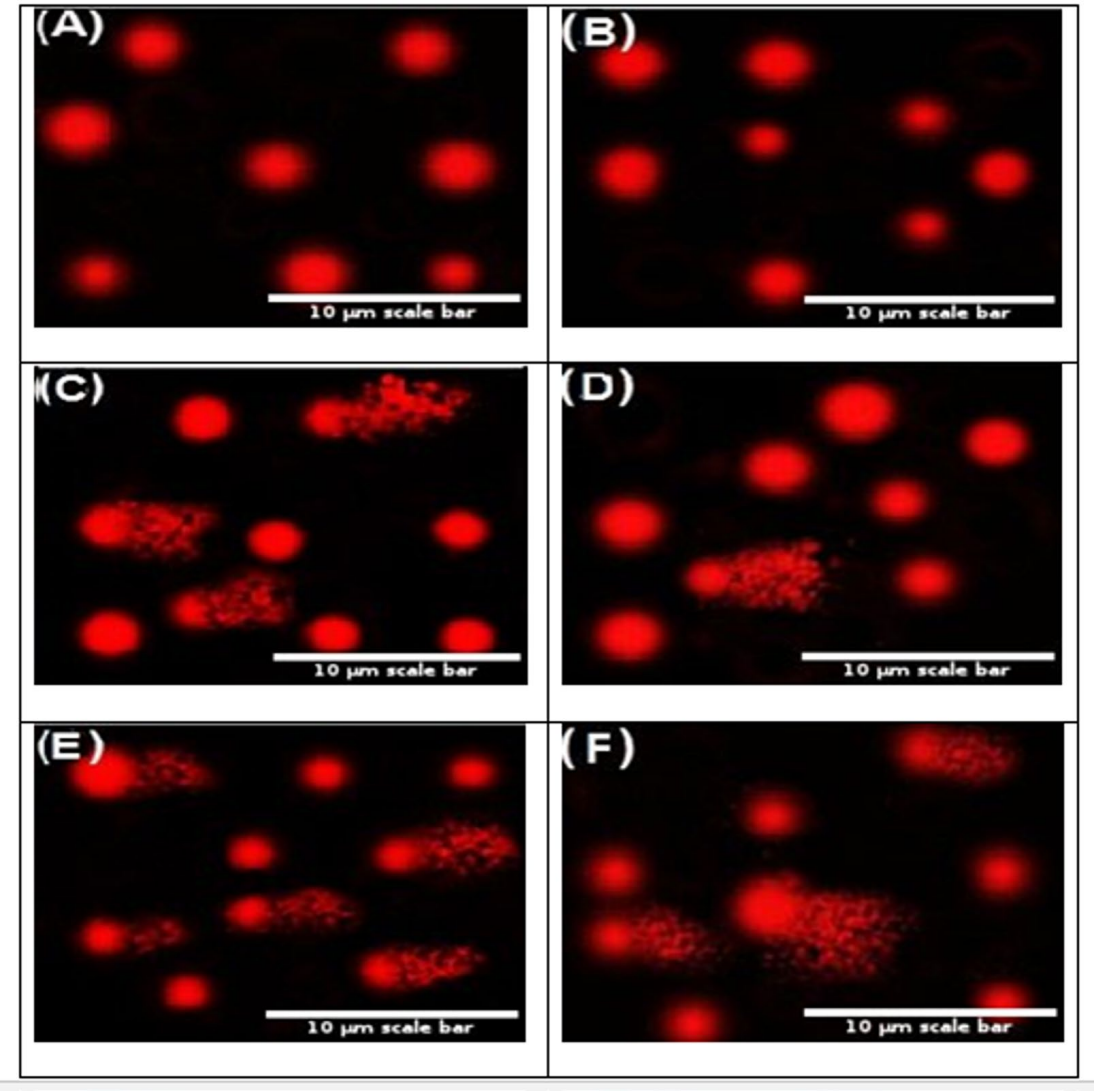
Standard comet test photos demonstrating DNA damage in YKO following exposure to various gold nanorod concentrations: A and B, which served as controls, and various doses in treated genes C (*CTR1* gene), D (*GYL1* gene), E (*RGM1* gene), and F (*MRPL24* gene). A fluorescent microscope with a 400× magnification was used to take the pictures. 10 μm is the scale bar.

Following treatment with gold nanorods at concentrations of 0.5, 1.0, and 1.5 mg/25 mL, DNA damage in *Salmonella typhimurium*,* Escherichia coli*, and *Saccharomyces cerevisiae* knockout strains was assessed using the comet assay. All treated groups exhibited significant increases in genotoxic indicators including tail length, tail DNA%, and tail moment compared with their respective untreated controls (*P* < 0.05), demonstrating dose-dependent DNA fragmentation (Figs. 1, 2 and 3).

### Comet assay’s genotoxic impact on *Salmonella* strains of various gold nanorods

All three tested concentrations of gold nanorods 0.5 mg/25 mM (S1), 1 mg/25 mM (S2), and 1.5 mg/25 mM (S3) showed genotoxic action. Concentration determined the degree of DNA damage, with S3 causing the most noticeable genotoxic effects, S2 having mild effects, and S1 having the least amount of genotoxicity (Table 4).

The percentage of tailed nuclei in all treated *Salmonella typhimurium* samples significantly increased as compared to the control group, suggesting increased DNA damage. Comet assay analysis confirmed this finding, as shown in Fig. 1, which showed a significant rise in DNA fragmentation in the treated groups. The concentration threshold at which the number of comets doubled to objectively evaluate the assay’s sensitivity.

The genotoxic potential of the AuNRs was confirmed by the comet frequency exceedingly twice that of the control at all tested doses of gold nanorods. Furthermore, DNA damage was made worse by treating with 25 mM of gold nanorods after pre-treating with doses of 0.5 mg/25 mM, 1.0 mg/25 mM, and 1.5 mg/25 mM. The observed genotoxicity was dose-dependent, as evidenced by the increasing increases in the proportion of tailed cells, tail length, tail DNA%, and tail moment with concentration. Statistically significant variances at *P* < 0.05 are shown by different superscript letters (a–d). Together, these results demonstrate that *Salmonella typhimurium* suffers substantial DNA damage from gold nanorods, and there is a direct link between concentration and genotoxic reaction.

**Table 4:** Following treatment with gold nanorods, shows the comet assay parameters derived from image analysis in cells of all groups.

Group	Tailed %	Untailed %	Tail length (µm)	Tail DNA (%)	Tail moment
Control	1.75	98.25	1.26 ± 0.10^d^	1.40 ± 0.08^d^	1.77 ± 0.12^d^
0.5	8.25	91.75	2.72 ± 0.12^c^	3.06 ± 0.15^c^	8.32 ± 0.45^c^
1	15.00	85.00	4.11 ± 0.35^b^	5.13 ± 0.20^b^	21.08 ± 1.15^b^
1.5	30.50	69.50	8.34 ± 0.55^a^	7.11 ± 0.30^a^	59.30 ± 2.80^a^

Using Duncan’s approach, different superscript letters in the same tail length column indicated a significant difference at *P* < 0.05.

### Impact of various gold nanorods on *E. coli* strains that are genotoxic

In *Escherichia coli*, gold nanorods showed definite genotoxic action at the three tested concentrations: 0.5 mg/25 mM (E1), 1.0 mg/25 mM (E2), and 1.5 mg/25 mM (E3). It was discovered that the degree of DNA damage varied with dose, with E3 causing the most genotoxicity, E2 causing moderate damage, and E1 causing the least amount of genotoxicity (Table 5).

The percentage of tailed nuclei in all treated *E. coli* cultures was much higher than in the untreated control, suggesting increased DNA fragmentation. Figure  2, which unequivocally displays a greater number of comets in the treated groups, further supported this increase in DNA damage. We employed a sensitivity metric based on the concentration at which the number of comets doubled in comparison to the control in order to improve the accuracy of comparison between treatment groups. This threshold was exceeded by all three tested concentrations, demonstrating that AuNRs cause significant DNA damage in *E. coli* cells. Furthermore, after being pre-treated with 0.5 mg/25 mM, 1.0 mg/25 mM, and 1.5 mg/25 mM, cells subjected to 25 mM AuNRs showed increased genotoxic effects, confirming the AuNR’s capacity to damage DNA. Higher amounts of AuNRs markedly enhanced the quantitative metrics determined from the comet assay tail length, percentage of tail DNA, and tail moment, highlighting the AuNRs’s dose-responsive genotoxicity. Statistically significant differences at *P* < 0.05 are shown by different superscript letters (a–d).

These results confirm the capacity of AuNRs to damage DNA in prokaryotic systems by strongly indicating that they have a concentration-dependent genotoxic effect on *E. coli*.

**Table 5:** Following treatment with a gold nanorod, comet assay parameters were determined by image analysis in cells from all groups.

Group	Tailed %	Untailed %	Tail length (µm)	Tail DNA (%)	Tail moment
Control	1.50	98.50	1.21 ± 0.08^d^	1.36 ± 0.06^d^	1.65 ± 0.10^d^
0.5	6.95	93.05	2.32 ± 0.25^c^	2.33 ± 0.12^c^	5.41 ± 0.30^c^
1	11.50	88.50	3.98 ± 0.28^b^	3.41 ± 0.18^b^	13.57 ± 0.65^b^
1.5	24.00	76.00	8.37 ± 0.79^a^	6.80 ± 0.25^a^	56.92 ± 2.10^a^

Using duncan’s approach, different superscript letters in the same tail length column indicated a significant difference at *P* < 0.05.

### YKO strains of various gold nanorods’ genotoxic impact using the comet assay

According to comet test data compiled in Table 6, gold nanorods demonstrated notable genotoxic effects in YKO strains at a dose of 1 mg/25 mM. Elevated levels of comet tail characteristics indicated that all examined YKO strains had significantly more DNA damage than the control group. Visual evidence of significant DNA fragmentation in treated yeast cells, as shown in Fig. 3, further confirmed the genotoxicity. We examined four YKO strains that were all lacking in one of the following genes: *CTR1*, *GYL1*, *RGM1*, and *MRPL24* in order to evaluate test sensitivity and genotoxic response across various genotypes. These genes were chosen because they act similarly to human genes linked to stress or apoptosis. All of the knockout strains had far larger percentages of tailed nuclei, longer tail lengths, higher tail DNA percentages, and more tail moments than the control, according to the comet assay. The *GYL1* mutant displayed the least amount of DNA fragmentation, which was nonetheless statistically significant, while the RGM1 deletion mutant revealed the most severe DNA damage, followed by *MRPL24* and *CTR1* knockouts. The sensibility threshold was set as the concentration at which the quantity of comets in the treated cells was at least twice that of the untreated control in order to offer an exact comparison measure. This threshold was surpassed by all four knockout strains, demonstrating their increased susceptibility to genotoxicity caused by AuNRs. Statistically significant differences at *P* < 0.05 are shown by different superscript letters (a–c).

These results unequivocally show that AuNRs cause substantial and genotype-dependent DNA damage in yeast knockout strains, underscoring the value of YKO models in assessing the genotoxicity of nanomaterials and determining gene-specific sensitivities.

**Table 6:** Comet assay parameters derived from imaging analysis of cells in each group following gold Nanorod treatment.

Group	Tailed %	Untailed %	Tail length (µm)	Tail DNA (%)	Tail moment
Control	2.50	97.50	1.44 ± 0.11^c^	1.52 ± 0.07^c^	2.19 ± 0.12^c^
*CTR1*	18.00	82.00	6.93 ± 0.53^b^	5.00 ± 0.20^b^	34.65 ± 1.85^b^
*GYL1*	7.40	92.60	2.38 ± 0.30^c^	2.36 ± 0.10^c^	5.62 ± 0.35^c^
*RGM1*	29.25	70.75	8.85 ± 0.42^a^	7.67 ± 0.28^a^	67.88 ± 2.25^a^
*MRPL24*	22.00	78.00	7.15 ± 0.40^a^	5.93 ± 0.22^a^	42.40 ± 1.90^a^

Using duncan’s approach, different superscript letters in the same tail length column indicated a significant difference at *P* < 0.05.

### Predicting protein-protein interactions using genemania

**Fig. 4:**
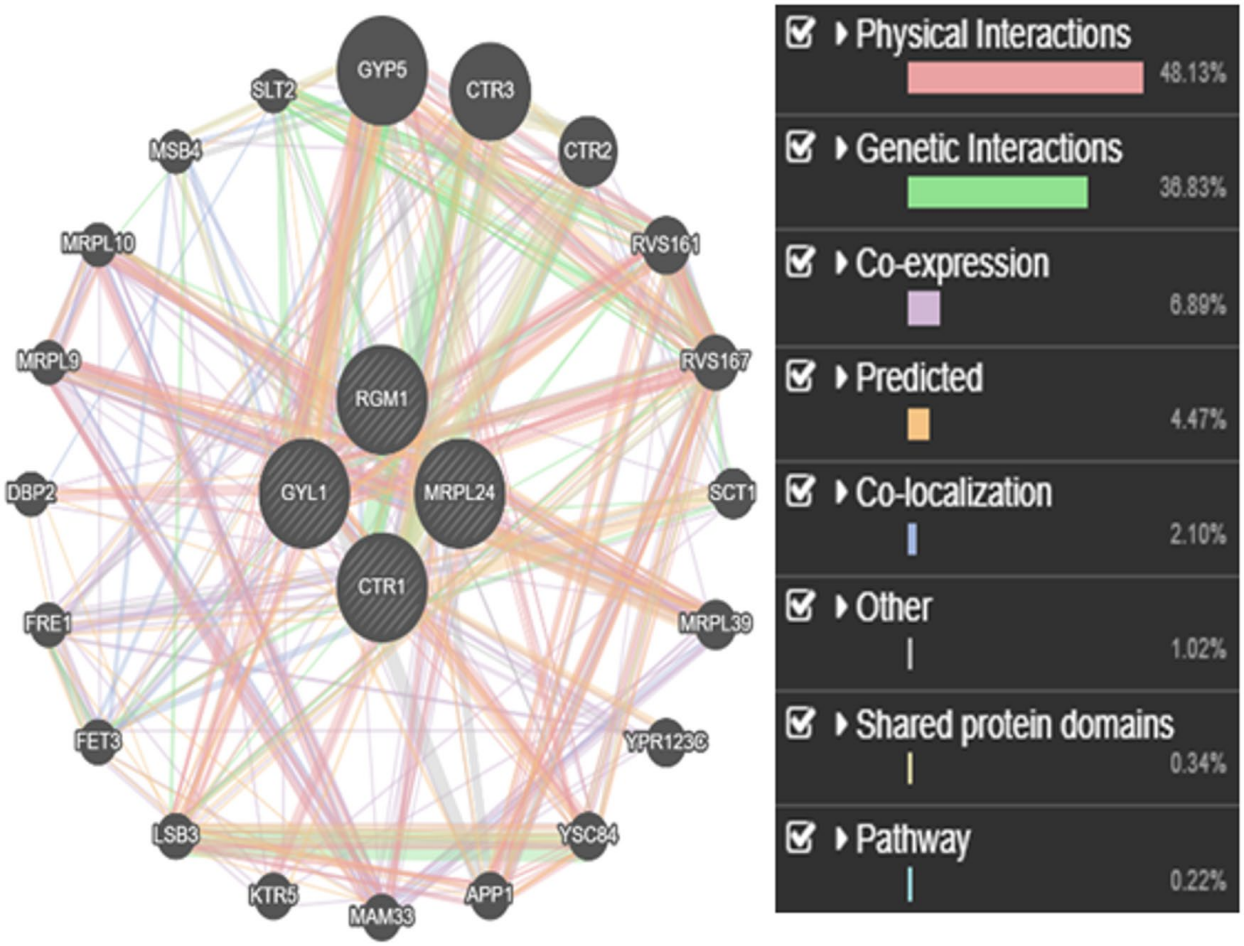
Shows the default query for the yeast cell cycle with all of its default parameters.

Using the GeneMANIA platform to predict protein–protein interactions (PPIs) further assisted the identification of important genes involved in the genotoxic response of yeast strains (Fig. 4). The platform enables thorough functional association analysis and incorporates several datasets pertinent to *Saccharomyces cerevisiae*. Every dataset that is analyzed adds to the total predictive value, allowing for a more thorough comprehension of gene function and interaction networks.

Four particular genes of interest in *S. cerevisiae* were analyzed using GeneMANIA. Using the provided query list as a guide, the tool produced unique functional networks that highlighted linked genes and pathways. A comprehensive web of biological relationships, including shared protein domains, co-expression, genetic linkages, and physical interactions, was exposed by these networks. GeneMANIA produced a distribution of interaction types, which included:

Co-expression: 6.89%, genetic interactions: 36.83%, and physical interactions: 48.13% Pathway associations: 0.22%, shared protein domains: 0.34%, other categories: 1.02%, co-localization: 2.10%, and predicted interactions: 4.47%.

These networks of interactions show how a wide range of related genes and biological processes are connected to yeast genes when they are searched.

Integrity in the decoding of topological properties was guaranteed by using the default network weighting algorithm and customizing the query parameters (e.g., setting network weight at 0.59%). Crucially, this method not only advances our knowledge of yeast gene function but also sheds light on conserved molecular mechanisms that might be pertinent to human disease pathways, especially those involving genes linked to cancer. The idea of functional homology and the usefulness of yeast as a model for researching genotoxic stress are supported by this cross-species conclusion.

These results are in line with those of Farley et al. (2010)^30^, who found that in yeast and mouse models, GeneMANIA performs better than or is on par with existing gene function prediction methods in terms of predictive accuracy. Both systems genetics researchers and molecular biologists can benefit from the platform’s extensive database, user-friendly interface, and high accuracy.

### In HepG2 cells, gold nanorods caused genotoxicity of a few associated genes, including *P53*,* Bcl-2*, and *Bax*

**Fig. 5:**
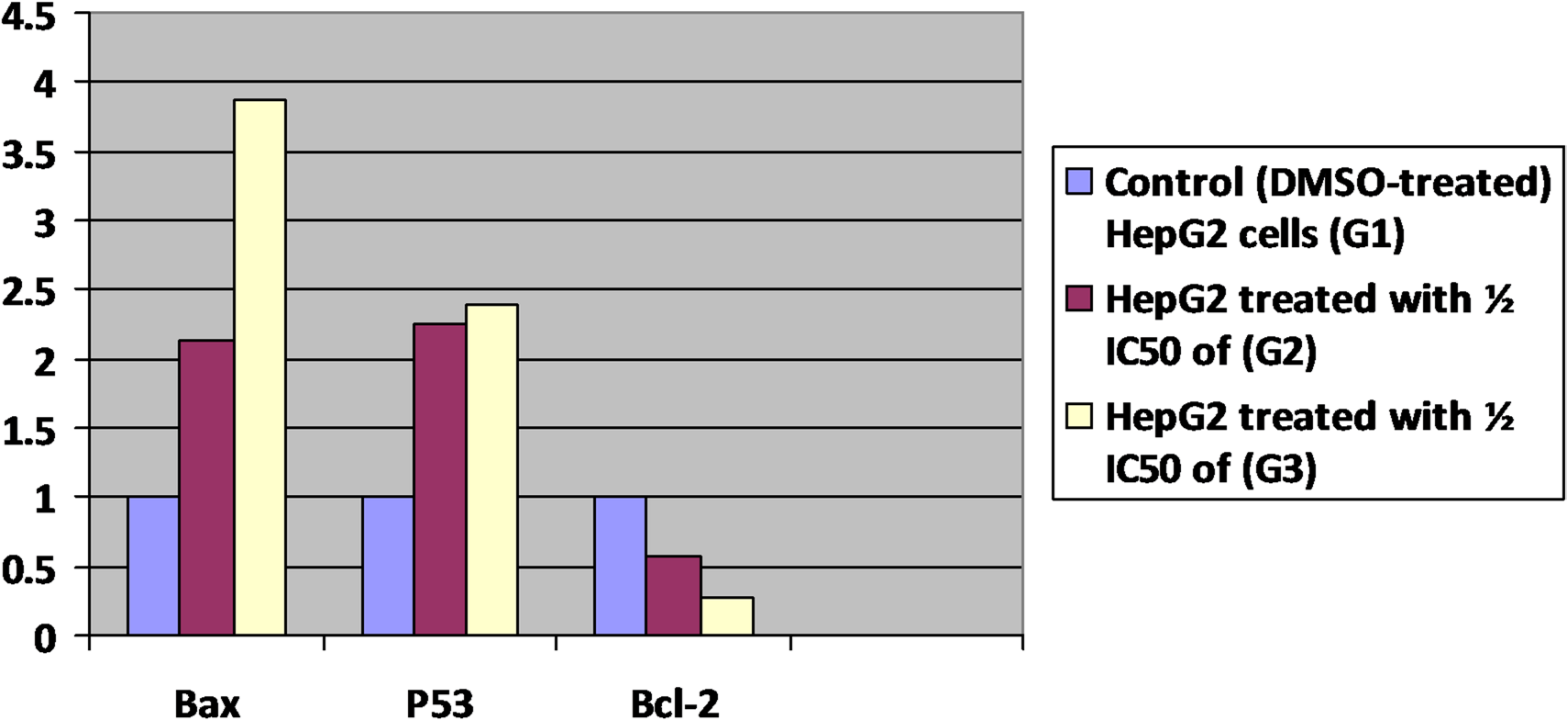
Quantification RT-PCR was used to evaluate the effects of 50 µg/mL AuNRs on apoptotic-related genes, including the mRNA expression of *Bax*,* Bcl-2*, and *p53*, with *P < 0.05 when compared to the control group.

It was investigated how apoptosis contributed to the cytotoxicity that gold nanorods caused in the HepG2 liver cancer cell lines. Real time PCR (qRT-PCR) was used to evaluate the expression levels of apoptosis-related genes in HepG2 cells, including *p53*,* Bcl-2*, and *Bax*. (Table 7) demonstrated that while the expression levels of the *Bcl-2* gene reduced, those of *p53* and *Bax* rose in comparison to the untreated group (control) (Fig. 5). These findings showed that the gold nanorods killed HepG2 cells by apoptosis, mostly through overexpression of the *p53* and *Bax* genes and downregulation of *Bcl-2*.

**Table 7:** Impact of administering gold Nanorod AuNRs on the relative expression of the *P53*,* Bcl-2*, and *Bax* genes in HepG2 cell lines using the RT-PCR method.

Sample data	Bax Aver CT	Δ Ct	ΔΔ Ct	Fold change	SEM
Control (DMSO-treated) HepG2 cells (G1)	22.81	0.00	0.00	1.00	0.05
HepG2 treated with ⅓ IC_50_ of (G2)	22.02	-1.10	-1.10	2.14	0.09
HepG2 treated with ½IC_50_ of (G3)	20.26	-1.95	-1.95	3.86	0.12

Sample data	*P53* Aver CT	Δ Ct	ΔΔ Ct	Fold change	SEM
Control (DMSO-treated) HepG2 cells (G1)	23.62	0.81	0.00	1.00	0.07
HepG2 treated with ½ IC_50_ of (G2)	22.76	-0.36	-1.17	2.25	0.12
HepG2 treated with ½ IC_50_ of (G3)	22.76	-0.45	-1.26	2.39	0.13

Sample data	*Bcl-2* Aver CT	Δ Ct	ΔΔ Ct	Fold change	SEM
Control (DMSO-treated) HepG2 cells (G1)	30.21	7.40	0.00	1.00	0.06
HepG2 treated with ½ IC_50_ of (G2)	31.3				
HepG2 treated with ½ IC_50_ of (G3)	32.44	9.23	1.83	0.28	0.01

**Fig. 6:**
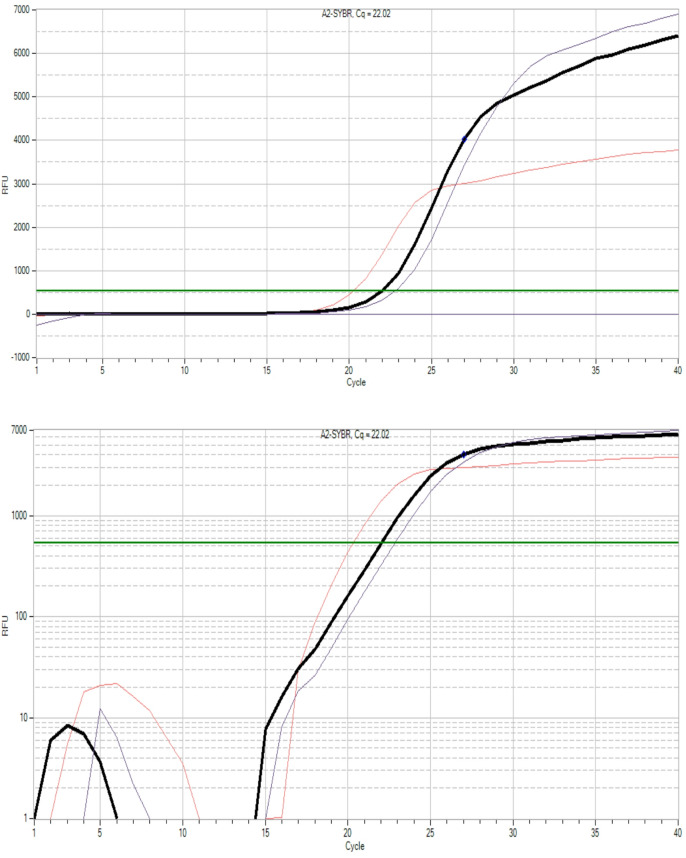
Linear (top) and logarithmic (bottom) amplification plots illustrating the Ct values of *Bax* gene expression in HepG2 cells after treatment with ½ IC_50_ concentrations of G2 and G3.

**Fig. 7:**
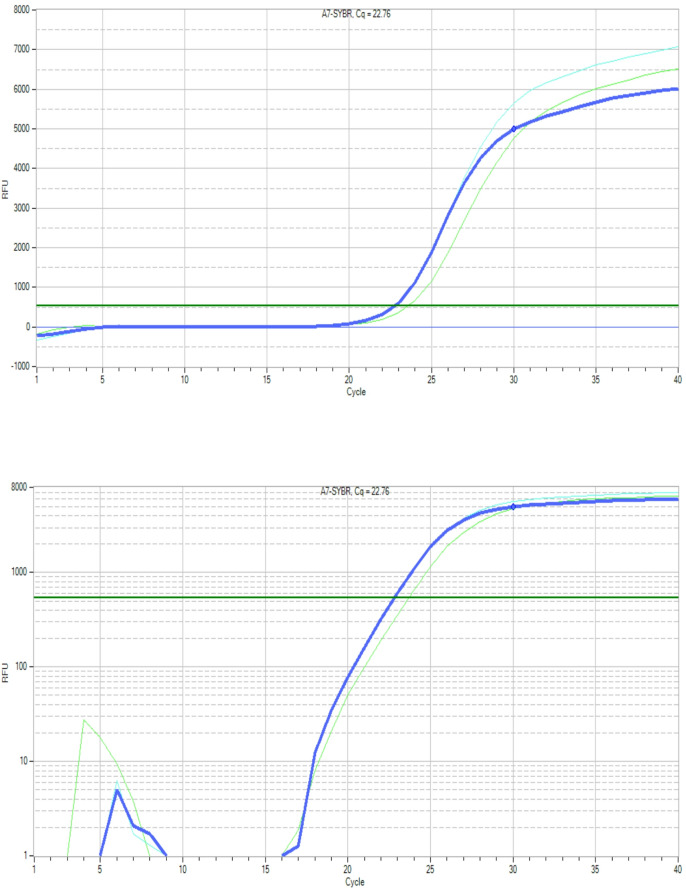
Linear (top) and logarithmic (bottom) amplification curves showing *p53* gene Ct values in HepG2 cells exposed to ½ IC_50_ doses of G2 and G3.

**Fig. 8:**
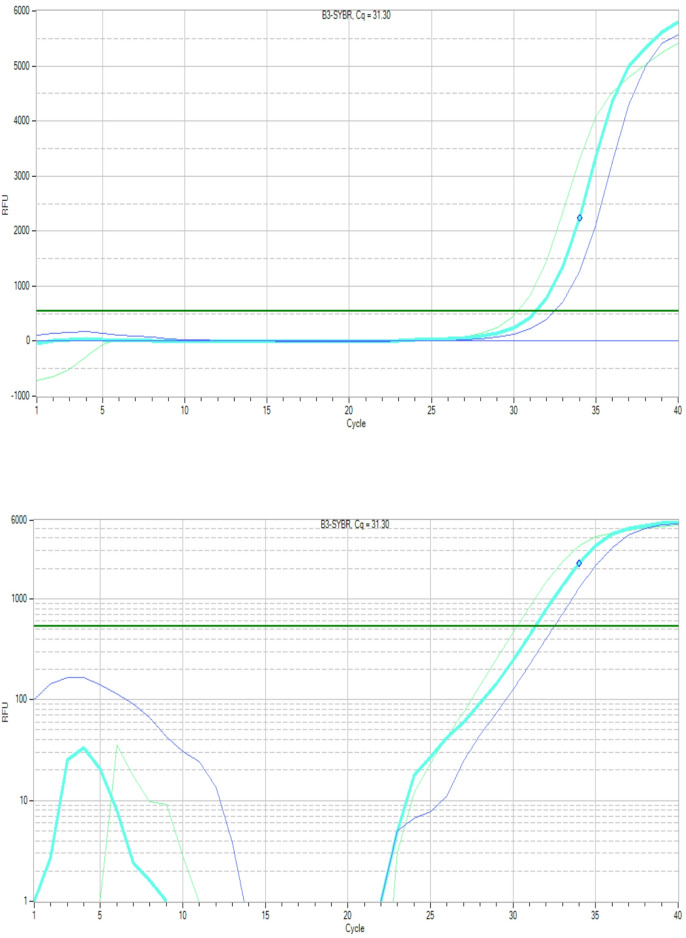
Amplification curves in both linear (top) and logarithmic (bottom) formats representing Ct values of *Bcl-2* gene in HepG2 cells following treatment with ½ IC_50_ concentrations of G2 and G3.

Figures 6 and 7, and 8 display the amplification curves (both linear and logarithmic views) for the expression of *Bax*,* p53*, and *Bcl-2* genes in HepG2 cells treated with half-maximal inhibitory concentrations (½ IC_50_) of G2 and G3. The Ct values observed in these figures indicate changes in gene expression following treatment. Specifically, *Bax* and *p53*, which are pro-apoptotic markers, showed increased expression, while *Bcl-2*, an anti-apoptotic gene, exhibited reduced expression in treated groups. These patterns suggest the activation of apoptotic pathways in response to G2 and G3 treatments, highlighting their potential cytotoxic effects on liver cancer cells.

At the conclusion of each qPCR run (60–95 °C), melting curve analysis was performed to ensure the absence of primer-dimer formation and the specificity of amplification. Primer specificity was confirmed by the one, distinct melting peak that each reaction produced.

## Discussion

The current study provides thorough proof of the genotoxic potential of AuNRs in a variety of biological models, such as human HepG2 liver cancer cells, *Salmonella typhimurium*,* Escherichia coli*, and YKO. The results support earlier studies on the toxicity of nanomaterials by offering a multifaceted knowledge of DNA damage caused by nanoparticles by combining prokaryotic and eukaryotic systems. Our investigation demonstrated a definite dose-dependent genotoxic effect of AuNRs in both bacterial strains using the comet assay, a proven technique for identifying DNA strand breaks^28,29^. *S. typhimurium* and *E. coli* showed a notable increase in tail length, tail DNA percentage, and tail moment at increasing concentrations, indicating enhanced DNA fragmentation, which is consistent with previous findings^12,30^. According to^25–27^, these results are consistent with earlier research on the genotoxic effects of metal ions and food additives on bacterial and eukaryotic systems.

Gene-specific reactions to AuNRs were very instructive in yeast models. The *RGM1* mutant displayed the most significant DNA damage among the four knockout strains (*CTR1*,* GYL1*,* RGM1*, and *MRPL24*), followed by *CTR1* and *MRPL24*. These results are consistent with earlier research assessing the genotoxic potential of food additives using the yeast comet assay^12^, which found increased DNA vulnerability upon ablation of stress-responsive genes. For genotoxicity testing, the yeast model has shown itself to be a sensitive and genetically tractable system that enables the discovery of conserved pathways that may be involved in oxidative stress, DNA repair, and apoptosis. The lack of a positive control in the comet experiment, such as cells treated with H2O₂, is one of the study’s limitations. Future research will include suitable positive controls to further establish assay sensitivity, even if the assay settings were validated in earlier studies^12,27^ and successfully detected DNA damage in treated samples.

The genotoxic effect of AuNRs was further confirmed by gene expression analysis in the HepG2 human liver cancer cell line. The anti-apoptotic gene *Bcl-2* was downregulated while pro-apoptotic genes *p53* and *Bax* were upregulated, indicating that intrinsic apoptotic pathways were activated in response to DNA damage caused by nanoparticles. These results are in line with our previous research, which demonstrated that doxorubicin and other cytotoxic substances, such as gold nanoparticles, cause comparable expression patterns and death in cancer cells^6,17,24^.

Furthermore, this apoptotic gene modulation is consistent with earlier findings on other metal compounds and nanoparticles, including cadmium chloride and zinc sulfate, which have also been demonstrated to impair DNA integrity and gene expression in yeast and human systems^26,27^. The predictive utility of apoptotic gene expression markers in evaluating genotoxic and cytotoxic effects is shown by the consistent patterns observed across these many chemical agents and models.

The GeneMANIA prediction server was used to analyze protein–protein interaction (PPI) networks in order to supplement experimental data. Their importance in stress and damage response networks was reinforced by the analysis, which found high functional correlations among the chosen yeast genes, with notable contributions from genetic interactions (36.83%) and physical interactions (48.13%). This method demonstrated that insights into human gene activity and possible cancer-related pathways can be consistently extrapolated from model organisms such as yeast^12,31^.

Consistent with earlier research that examined drug-induced cytotoxicity and nanomaterial cytotoxicity using functional genomics and bioinformatics tools^6,24,26,27^, this study shows the value of integrating in silico prediction platforms with experimental genotoxicity assays. In addition to offering mechanistic insights, these integrated approaches help create and regulate nanomaterials in clinical and industrial settings in a safer manner.

This paper offers a thorough evaluation of the genotoxic potential of AuNRs by the integrative use of bacterial, yeast, and mammalian models. Because they lack nuclear organization and have simpler repair mechanisms, prokaryotic systems like *Escherichia coli* and *Salmonella typhimurium* provide quick and sensitive detection of direct DNA damage. On the other hand, yeast functions as a eukaryotic model, offering insights into eukaryotic genotoxic mechanisms through conserved DNA repair and stress-response pathways that are similar to those in human cells. The haploid *S. cerevisiae* knockout strains were selected based on sequence homology to human cancer-related genes and their known roles in stress, DNA damage response, and mitochondrial function. Specifically:

- *CTR1* metal-ion homeostasis and oxidative stress.
- *GYL1* stress-related growth regulation.
- *RGM1* transcriptional regulation of stress/DNA-stability genes.
- *MRPL24* mitochondrial function and apoptosis.

These genes were chosen because they represent yeast functional analogs of human p53, Bax, and Bcl-2 pathways, enabling cross-species comparison of gold nanorod-induced genotoxicity. This has been clarified in the revised manuscript. Lastly, mammalian HepG2 cells offer a physiologically appropriate paradigm for assessing cellular stress responses and apoptosis-related gene expression. When combined, these alternative models increase the findings’ dependability and translational value by bridging the gap between simple DNA damage detection and intricate mammalian responses^3,8,12^.

Our findings imply that whereas AuNRs show encouraging pro-apoptotic activity in cancer cells, they also represent genotoxic hazards to non-target systems, which is relevant given the increased interest in nanotechnology for medicinal applications, particularly in targeted cancer therapy. Prior to clinical usage, this necessitates meticulous risk-benefit analysis, tailored delivery methods, and precise dosage management^6,26,27,32]^.

## Conclusion

The genotoxic potential of AuNRs in a variety of biological systems, such as human cell lines, YKO strains, and *E. coli*, is highlighted in this paper. The results demonstrate that AuNRs at concentrations of 0.5 mg/25 mM, 1 mg/25 mM, 1.5 mg/25 mM cause detectable DNA damage, with the degree of damage increasing dose-dependently, according to comet assay and protein-protein interaction analysis uses GeneMANIA. The YKO strains showed significant DNA fragmentation and tail moments, especially those lacking in genes like RGM1 and MRPL24, suggesting that these genes may be involved in DNA repair or damage response mechanisms.

Additionally, the use of methods for predicting protein-protein interactions, such GeneMANIA, revealed conserved biological pathways that may be impacted by nanomaterials and provided insights into the underlying molecular interactions. This method not only supported the actual findings from the genotoxicity tests but also deepened our understanding of the potential molecular effects of nanomaterials like AuNRs on cellular pathways.

The results are in line with other research on heavy metals and food additives that also showed cytotoxic and genotoxic effects in human cells and yeast models^17,25–27^. These findings highlight how crucial it is to use both experimental and computational techniques in order to thoroughly assess the biological effects of nanomaterials.

In summary, AuNRs clearly show genotoxic activity that is regulated by genetic background and concentration. These findings call for more research to clarify the mechanisms of action and long-term biological impacts of nanomaterials and highlight the necessity for their careful usage in biomedical and environmental applications.

## Data Availability

Data availabilityThe datasets and code generated and/or analyzed during the current study will be available from the corresponding author on reasonable request. Certain data supporting the findings of this study were obtained from Thermo Fisher Scientific, and restrictions apply to their availability. These datasets were used under license and are not publicly accessible. Access to these data may be granted by the authors upon reasonable request and with permission from Thermo Fisher Scientific. All experimental data were generated using microbial strains, yeast knockout models, and the human hepatocellular carcinoma cell line HepG2, none of which involve personally identifiable information. The HepG2 cell line was obtained from the American Type Culture Collection (ATCC), Manassas, Virginia, USA. Yeast deletion strains (YKO collection) were accessed via Thermo Fisher Scientific at: https://www.thermofisher.com/order/catalog/product/. Gene information and sequences were retrieved from the Saccharomyces Genome Database (SGD): https://www.yeastgenome.org. Protein-protein interaction networks were predicted using the GeneMANIA platform: http://www.genemania.org.
